# Machine learning-based nomogram for distinguishing between supratentorial extraventricular ependymoma and supratentorial glioblastoma

**DOI:** 10.3389/fonc.2024.1443913

**Published:** 2024-09-09

**Authors:** Ling Chen, Weijiao Chen, Chuyun Tang, Yao Li, Min Wu, Lifang Tang, Lizhao Huang, Rui Li, Tao Li

**Affiliations:** ^1^ Department of Radiology, Liuzhou Worker’s Hospital, Liuzhou, Guangxi, China; ^2^ Department of Radiology, The First Affiliated Hospital of Guangxi Medical University, Nanning, Guangxi, China; ^3^ Department of Neurosurgery, Liuzhou Worker’s Hospital, Liuzhou, Guangxi, China; ^4^ Department of Radiology, The Fourth Affiliated Hospital of Guangxi Medical University, Liuzhou, China

**Keywords:** machine learning, nomogram, glioblastoma, ependymoma, magnetic resonance imaging

## Abstract

**Objective:**

To develop a machine learning-based nomogram for distinguishing between supratentorial extraventricular ependymoma (STEE) and supratentorial glioblastoma (GBM).

**Methods:**

We conducted a retrospective analysis on MRI datasets obtained from 140 patients who were diagnosed with STEE (n=48) and GBM (n=92) from two institutions. Initially, we compared seven different machine learning algorithms to determine the most suitable signature (rad-score). Subsequently, univariate and multivariate logistic regression analyses were performed to identify significant clinical predictors that can differentiate between STEE and GBM. Finally, we developed a nomogram by visualizing the rad-score and clinical features for clinical evaluation.

**Results:**

The TreeBagger (TB) outperformed the other six algorithms, yielding the best diagnostic efficacy in differentiating STEE from GBM, with area under the curve (AUC) values of 0.735 (95% CI: 0.625-0.845) and 0.796 (95% CI: 0.644-0.949) in the training set and test set. Furthermore, the nomogram incorporating both the rad-score and clinical variables demonstrated a robust predictive performance with an accuracy of 0.787 in the training set and 0.832 in the test set.

**Conclusion:**

The nomogram could serve as a valuable tool for non-invasively discriminating between STEE and GBM.

## Introduction

Ependymoma accounts for 5% of primary central nervous system (CNS) tumors (1). It exhibits a predilection for specific age-location preferences, commonly observed in the posterior fossa among children and in the supratentorial and spine compartments among adults (2–4). In some cases, they may develop as STEE. The differential diagnosis of STEE poses a greater challenge compared to infratentorial ependymomas, as infratentorial ependymomas predominantly presents within the ventricles, while STEE may manifest in cortical regions outside the ventricles, resembling other high-grade tumors such as GBM. It is important to note that there are some similarities between STEE and GBM in terms of their overlapping clinical and routine MRI features, which can lead to diagnostic confusion. Despite the rarity of STEE, preoperative accurate differentiation is important, because the treatment and prognosis are totally different. Current guidelines advocate for total resection as the primary treatment approach for STEE, whereas GBM necessitates a combination of total resection, radiotherapy, and chemotherapy (5, 6). Therefore, achieving an accurate preoperative diagnosis holds paramount importance for both tumor types and significantly influences treatment selection and prognosis evaluation.

Advanced MRI techniques, such as perfusion MRI (7–9), magnetic resonance spectroscopy (MRS) (10–12), and diffusion-weighted imaging (DWI) (13–15) have demonstrated significant potential in the preoperative diagnosis of intracranial tumors. These non-invasive methods offer valuable insights into tumor vascularity, metabolic activity, and cellular proliferation. However, both STEE and GBM exhibit restricted diffusion and significantly hyperperfusion patterns, along with altered levels of glioma metabolites (15–18). Consequently, accurate differentiation between these two tumors remains a challenging due to their analogous tissue characteristics. While pathological diagnosis is currently one of the most widely employed and reliable approach for determining neurological tumor types and assessing their malignancy, it is imperative to acknowledge its inherent limitations and associated risks (19). First, the presence of highly heterogeneous tumors introduces potential sampling errors due to the wide diversity within tumor tissues. In addition, biopsy, as an invasive procedure, cannot rule out the risk of bleeding or infection complications (20).

Therefore, noninvasive assessment of the entire tumor *in vivo* could serve as a valuable adjunct to pathological diagnosis, aiding in therapy planning and prognostic prediction. Machine learning is an intriguing field that leverages advanced algorithms to unveil latent information embedded within medical images (21–23). The non-invasive nature of machine learning confers distinct advantages over invasive procedures such as biopsy or surgical resection, rendering them a secure alternative for patients who may not be suitable candidates for surgery due to underlying medical conditions or the presence of lesions in critical brain regions. Previous studies have demonstrated the significant role of machine learning in facilitating the identification of distinct subtypes of brain tumors (24, 25), predicting tumor genotyping (26), and assessing prognosis (27). These studies offer a machine learning-based theoretical framework that can potentially facilitate the discrimination between STEE and GBM. Despite the promising potential of machine learning algorithms in medical diagnostic, the utility of machine learning-based nomograms in distinguishing between these two tumors remains uncertain and further investigation is warranted. Therefore, this study aims to develop a nomogram based on multi-parameter MRI machine learning that can effectively classify STEE and GBM. To the best of our knowledge, there is a paucity of research on nomogram based on machine learning in patients with STEE and GBM, and our study adds to the body of knowledge in this area.

## Materials and methods

### Patients

**Figure 1:**
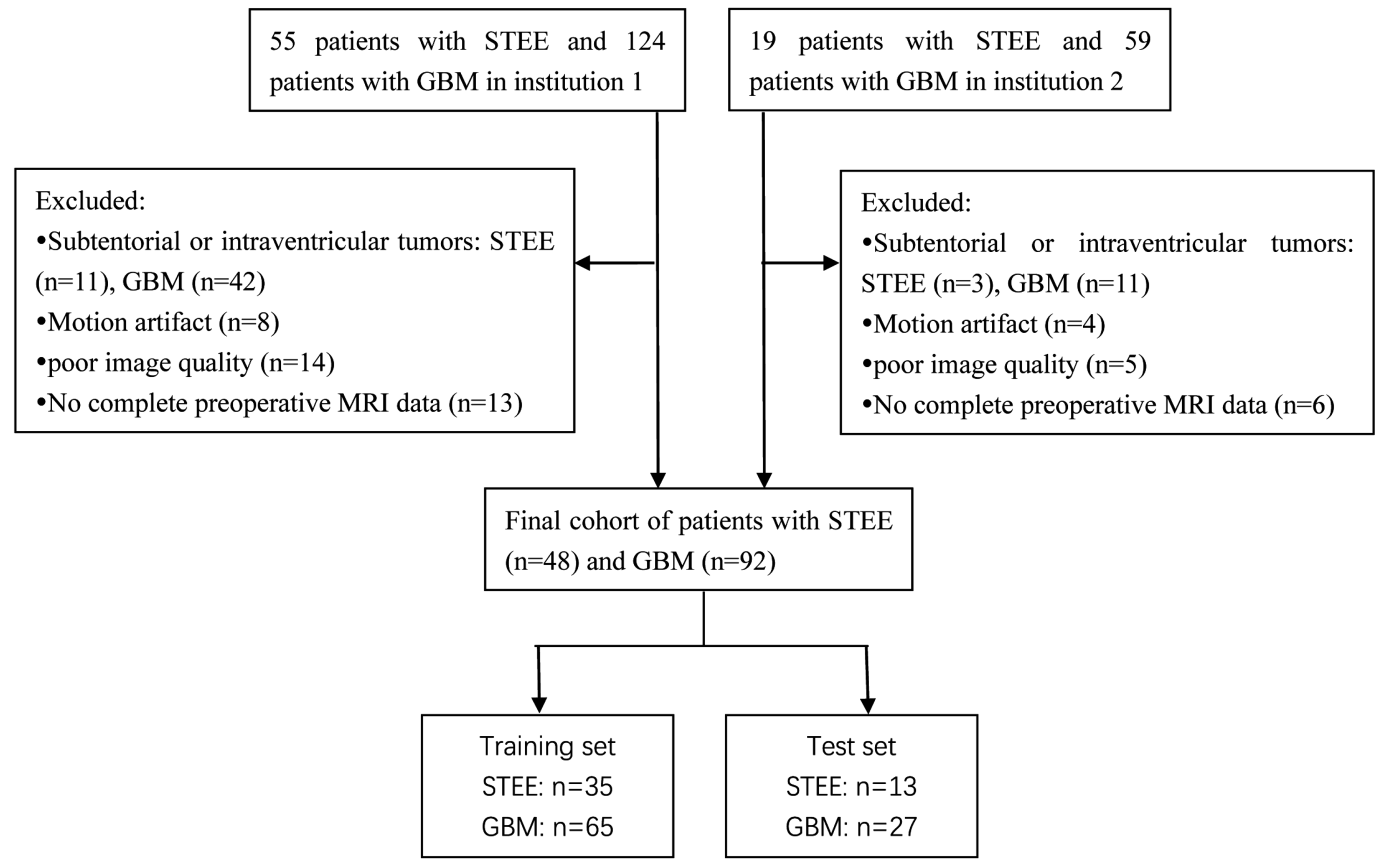
The patient selection flow chart.

This retrospective study was approved by the institutional research ethics review board, and the requirement for obtaining patient consent was waived. The present study retrospectively enrolled a total of 183 patients diagnosed with GBM and 74 patients diagnosed with STEE from two participating institutions in our cohort. Institution 1 recruited patients between January 2016 and December 2023, while institution 2 recruited patients between January 2018 and December 2023. The clinical data on various parameters, including age, gender, tumor size, preoperative Karnofsky Performance Status (KPS) score, and lateral ventricle involvement was extracted from both the hospital information system and the Picture Archiving and Communication Systems (PACS). All patients included in this study had undergone surgical resection, and the final diagnosis was confirmed through histopathological examination. The ventricular tumor was identified through MRI, with the primary mass predominantly located within the ventricle. Extra-ventricular tumors are characterized by their main body being situated in the external cerebral parenchyma adjacent to the ventricle, allowing for contact with the lateral ventricle. The inclusion criteria were as follows: histopathologic diagnosis of ependymomas or GBM; the tumor was supratentorial and outside the lateral ventricle, confirmed by MRI; all preoperative MRI performed before any intervention; conventional MR images, including unenhanced T1- and T2-weighted images and contrast-enhanced T1-weighted images, were available. Exclusion criteria included subtentorial or intraventricular tumors, motion artifacts, and poor image quality. The flowchart illustrating the process of patient selection is presented in **Figure 1**.

### MRI protocol

MRI imaging data included axial T1WI, T2WI and Gd-T1WI sequences obtained on two 1.5 MRI system (GE, Octane, United States; Siemens, Altea, Germany) and two 3.0T MRI system (Philips, Achieva, Netherlands; GE, Premier, United States). The sequences were acquired with a field of view (FOV) measuring 512×512 mm, a slice thickness of 5/3.0/2.5 mm, and a matrix size of 256×217/256×203/256×192. T1-weighted images were obtained using a repetition time (TR) of 2000 ms and an echo time (TE) of 19 ms, while the acquisition parameters for T2-weighted images included variable TRs ranging from 2600 to 5844 ms and TEs ranging from 80 to 129 ms. Gadolinium-enhanced T1-weighted images were acquired following intravenous injection of gadoterate meglumine through the median cubital vein at a flow rate of 2 mL/second (0.2 mL/kg body weight).

### Radiomics process

#### Images preprocessing and segmentation

**Figure 2:**
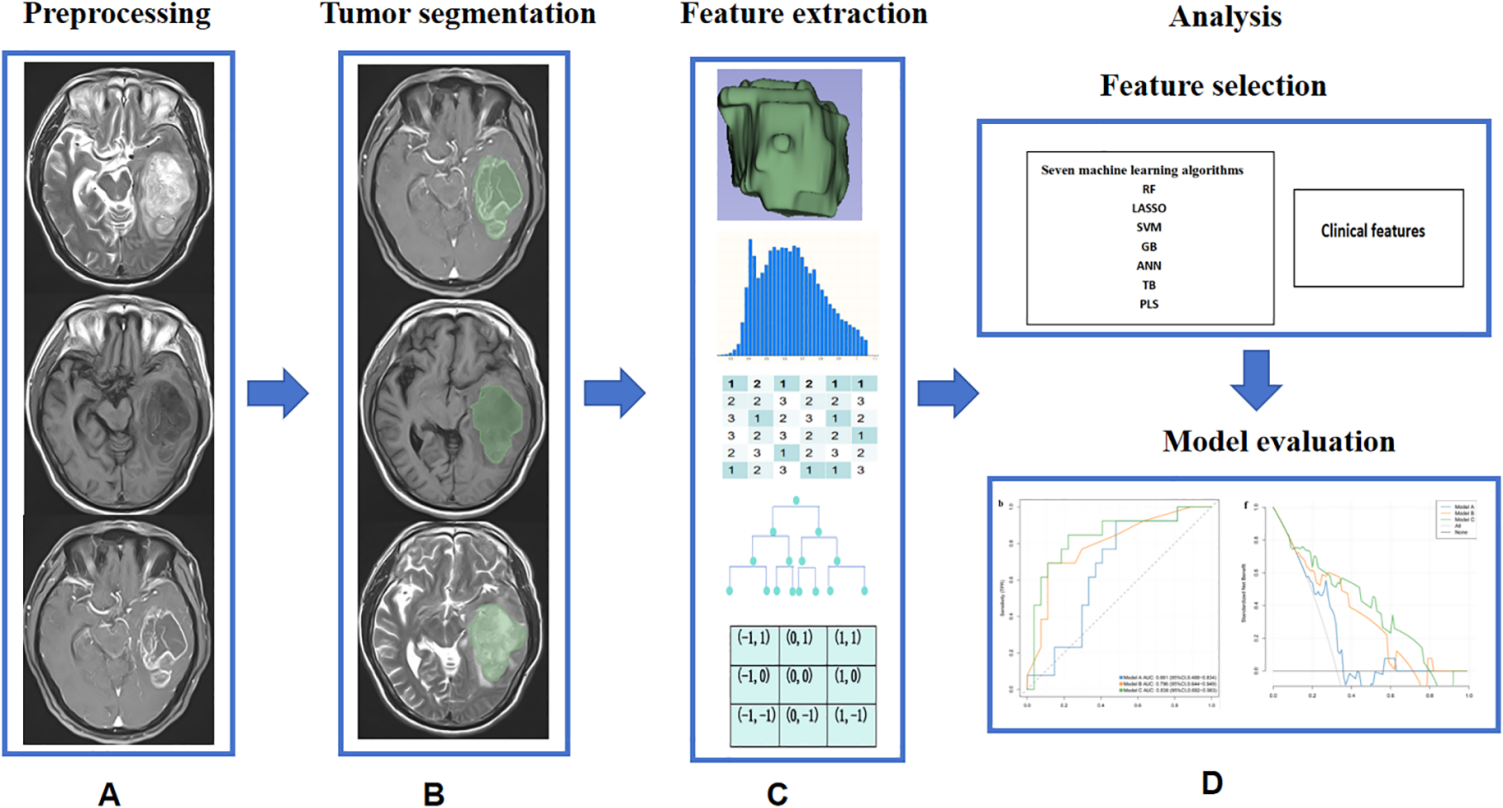
Machine learning pipeline for distinguishing between STEE and GBM. **(A)** Image preprocessing; **(B)** Tumor segmentation; **(C)** The feature extraction algorithm used in this study; **(D)** Model construction and evaluation.

The pre-processing of MRI images and radiomics pipeline is illustrated in **Figure 2**. Firstly, Gd-T1WI, T2WI and T1WI DICOM images were imported into the 3D Slicer software (version 5.3.0; https://www.slicer.org/). Subsequently, the images underwent resampling to achieve a voxel size of 1mm×1mm×1 mm while discretizing the gray levels with a bin width of 25. Secondly, we employed a semi-automatic approach for tumor segmentation. Specifically, each Gd-T1WI slice was segmented slice by slice along the enhanced tumor edge. Following automatic registration processing, the segmentation results were matched with T2WI and T1WI images. The task of tumor segmentation was independently performed by two neuroradiologists possessing over a decade of experience in this field. Excellent agreement between observers was indicated by interclass correlation coefficient values ranging from 0.75 to 1. Any discrepancies between the two neuroradiologists were resolved through consensus.

### Feature extraction

Radiomics feature extraction was performed using FeAture Explorer (https://github.com/salan668/FAE, version 0.3.7) in Python (version 3.7.6). We extracted a total of 2,553 features for each patient, consisting of shape features (n=14); first-order features (n=18); texture features including gray level co-occurrence matrix (GLCM, n=24), gray level dependence matrix (GLDM, n=14), gray level run length matrix (GLRLM, n=16), gray level size zone matrix (GLSZM, n=16), and neighborhood gray-tone difference matrix (NGTDM, n=5); wavelet transform (n=744) and Laplacian of Gaussian filter (n=93).

#### Feature selection and signature development

The datasets were randomly partitioned into a training set and a test set in a 7:3 ratio. Prior to feature reduction and selection in the training cohort, all extracted features underwent Z-score normalization for standardization purposes. The discriminative potential of relevant features between STEE and GBM at baseline was assessed using either the Mann-Whitney *U*-test or independent *t*-test. Subsequently, seven machine learning algorithms were employed: the support vector machine (SVM), random forest (RF), and the least absolute shrinkage and selection operator (LASSO), Artificial Neural Networks (ANN), TreeBagger (TB), Gradient Boosting (GB) and Partial Least-squares (PLS). These algorithms were compared to determine the optimal algorithm for constructing signatures. A machine learning nomogram combining the rad-score and clinical variables was constructed using multivariate logistic regression analysis. The nomogram was validated using the test cohort. The performance of the predictive model was evaluated using various metrics including the AUC, accuracy, sensitivity, specificity, positive predictive value (PPV), and negative predictive value (NPV). The DeLong test was utilized to compare the performance of the ROC curves. The calibration curve was employed to assess the level of concordance between the predicted probability and observed outcomes across various risk levels. The Hosmer-Lemeshow test was conducted to evaluate the goodness-of-fit for all models. Decision curve analysis (DCA) was executed to quantify the net benefits at different threshold probabilities in the test set.

### Statistical analysis

SPSS (version 27.0; IBM) and R statistical software (version 4.0.2) were used for statistical analyses. The independent-samples *t*-test for continuous variables and Mann-Whitney *U*-test was used for categorical variable. *P* values < 0.05 were considered indicative of statistical significance. Data were presented as mean ± SD for continuous variables and as frequency (%) for categorical variables.

## Results

### Feature Selection and Machine Learning Model Construction

The baseline characteristics of the patients are displayed in **Table 1**. The baseline analysis excluded 2,085 out of the initially extracted 2,553 features, resulting in a refined set of only 468 features for advanced variable screening. Finally, the pipeline generated a more robust model consisting of nine features. Among the nine most contributing features, four were extracted from Gd-T1WI (including wavelet-HHH_firstorder_Entropy, wavelet-LHH_gldm-HighGraylevelEmphasis, Wavelet-HLL_glcm_JointAverage, and wavelet-LLL-glszm_GraylevelNonUniformity), three from T2WI (including original_glrlm_RunLengthNonUniformity, wavelet-HHH_firstorder_Variance, and wavelet-HHH_glszm_SmallAreaHighGraylevelEmphasis), and two from T1WI (including wavelet-HHH_glcm-Autocorrelation and wavelet-LHH_glrlm_RunLengthNonUniformityNormalized).

**Table 1:** Patient’s characteristics at baseline.

Variables	Total	STEE	GBM	*P value*
No. of patients	140	48	92	/
Age(years)	45.16 ± 10.66	47.18 ± 9.92	41.27 ± 11.06	0.002
Gender(female)	66(47.1%)	22(45.8%)	44(47.8%)	0.823
Tumor size	108.15 ± 51.16	116.91 ± 57.87	91.36 ± 28.60	0.005
KPS	72.21 ± 14.50	70.76 ± 14.54	75.00 ± 14.14	0.101
Ventricle involvement	30(21.4%)	13(27.1%)	17(18.5%)	0.239

GBM, glioblastoma; STEE, supratentorial extraventricular ependymomas; KPS, karnofsky performance status.

The comparison of RF, LASSO, SVM, ANN, GB, TB and PLS algorithms on both the training and test sets is presented in **Table 2**. The results demonstrated that the TB algorithm exhibited superior performance on both the training set (AUC=0.735, 95%CI: 0.624-0.844) and test set (AUC=0.796, 95%CI: 0.644-0.949), surpassing six other algorithms.

**Table 2:** Performance evaluation of seven models for distinguishing between STEE and GBM.

Models	AUC	95% CI	Cutoff	Acc	Sen	Spe	PPV	NPV	Task
**RF**	0.714	0.593-0.836	0.275	0.790	0.714	0.831	0.694	0.844	Training
	0.691	0.474-0.907	0.275	0.750	0.615	0.815	0.615	0.815	Test
**LASSO**	0.512	0.377-0.646	-1.062	0.680	0.286	0.892	0.588	0.699	Training
	0.593	0.400-0.785	-1.062	0.525	0.692	0.444	0.375	0.750	Test
**SVM**	0.516	0.388-0.644	0.715	0.620	0.371	0.754	0.448	0.690	Training
	0.593	0.393-0.792	0.715	0.650	0.462	0.741	0.462	0.741	Test
**ANN**	0.515	0.405-0.624	0.324	0.680	0.114	0.985	0.800	0.674	Training
	0.506	0.351-0.660	0.324	0.675	0.000	1.000	–	0.675	Test
**GB**	0.726	0.611-0.841	0.005	0.770	0.714	0.800	0.658	0.839	Training
	0.692	0.501-0.883	0.005	0.775	0.692	0.815	0.643	0.846	Test
**TB**	0.735	0.625-0.845	0.300	0.760	0.629	0.831	0.667	0.806	Training
	0.796	0.644-0.949	0.300	0.825	0.692	0.889	0.750	0.857	Test
**PLS**	0.583	0.462-0.704	0.416	0.700	0.229	0.954	0.727	0.697	Training
	0.561	0.375-0.747	0.416	0.675	0.077	0.963	0.500	0.684	Test

Lasso, The least absolute shrinkage and selection operator; SVM, Support Vector Machine; RF, random forest; AUC, the area under the receiver operator characteristics curve; CI, confidence interval; PPV, positive predictive value; NPV, negative predictive value.

### Clinical Model Construction

To establish a predictive clinical model for distinguishing STEE and GBM, the development of the clinical model necessitates both univariate regression analysis and multivariate regression analysis to identify statistically significant factors. Univariate regression models focus on assessing the impact of individual variables on the dependent variable; however, they have a major limitation in not accounting for potential confounding factors. In contrast, multiple regression models consider multiple potential influencing factors, enabling more accurate evaluation of each factor’s independent influence on the dependent variable while controlling for other variables, thus yielding more reliable conclusions. The results of univariate and multivariate regression analysis of clinical variables to differentiate STEE patients from GBM patients are shown in **Table 3**. The results from **Table 3** indicated that age, tumor size, and rad-score exhibited statistically significant associations in the univariate regression analysis (*p*<0.05). The multivariate regression analysis revealed that only age and tumor size demonstrated statistically significant associations in differentiating STEE from GBM. Consequently, we incorporated these significant features (age and tumor size) to constructed a clinical model. The training set and test set AUC values for this clinical model were 0.69 and 0.684, respectively.

**Table 3:** Univariate and multivariate analyses of clinical variables and radiomics signature to differentiate STEE patients from GBM patients.

Variables	Univariate		Multivariate	
	OR (95%CI)	*P value*	OR (95%CI)	*P value*
Tumor size	0.988(0.979,0.997)	0.007	0.989(0.979, 1.000)	0.042
Age	0.947(0.913,0.981)	0.003	0.956(0.919,0.994)	0.023
KPS	1.022(0.996,1.048)	0.103	/	/
Gender(female)	0.923(0.458,1.858)	0.823	/	/
Ventrical involvement	1.639(0.717,3.743)	0.241	/	/
Rad-score	39.575(8.611,181.892)	<0.001	30.100(6.253,144.887)	<0.001

KPS, karnofsky performance status; CI, confidence interval.

### The performance of the nomogram

**Figure 3:**
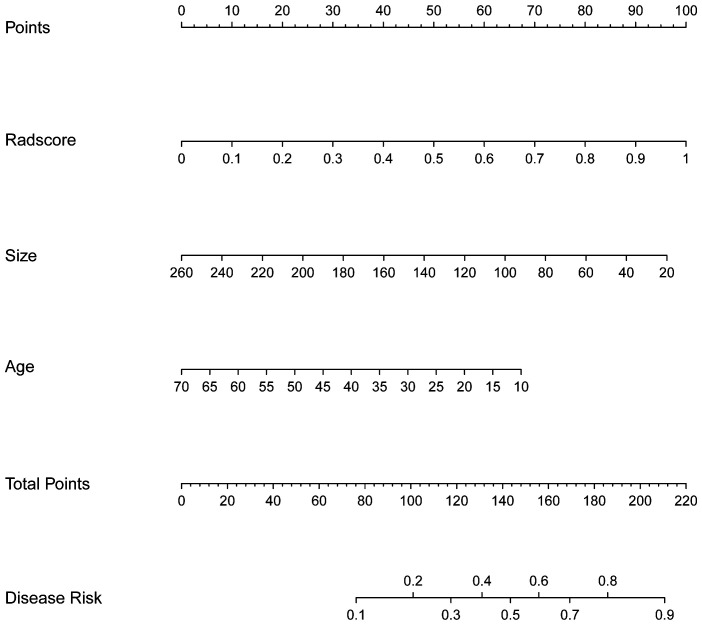
The nomogram used to distinguish STEE from GBM. For instance, in the case of a 60-year-old patient with a tumor size of 180mm^3^ and Radsore value of 0.5, the cumulative score should approximate to 94 scores, indicating a disease risk probability below 0.2. Based on these findings, it is highly probable that this individual is diagnosed with GBM.

**Figure 4:**
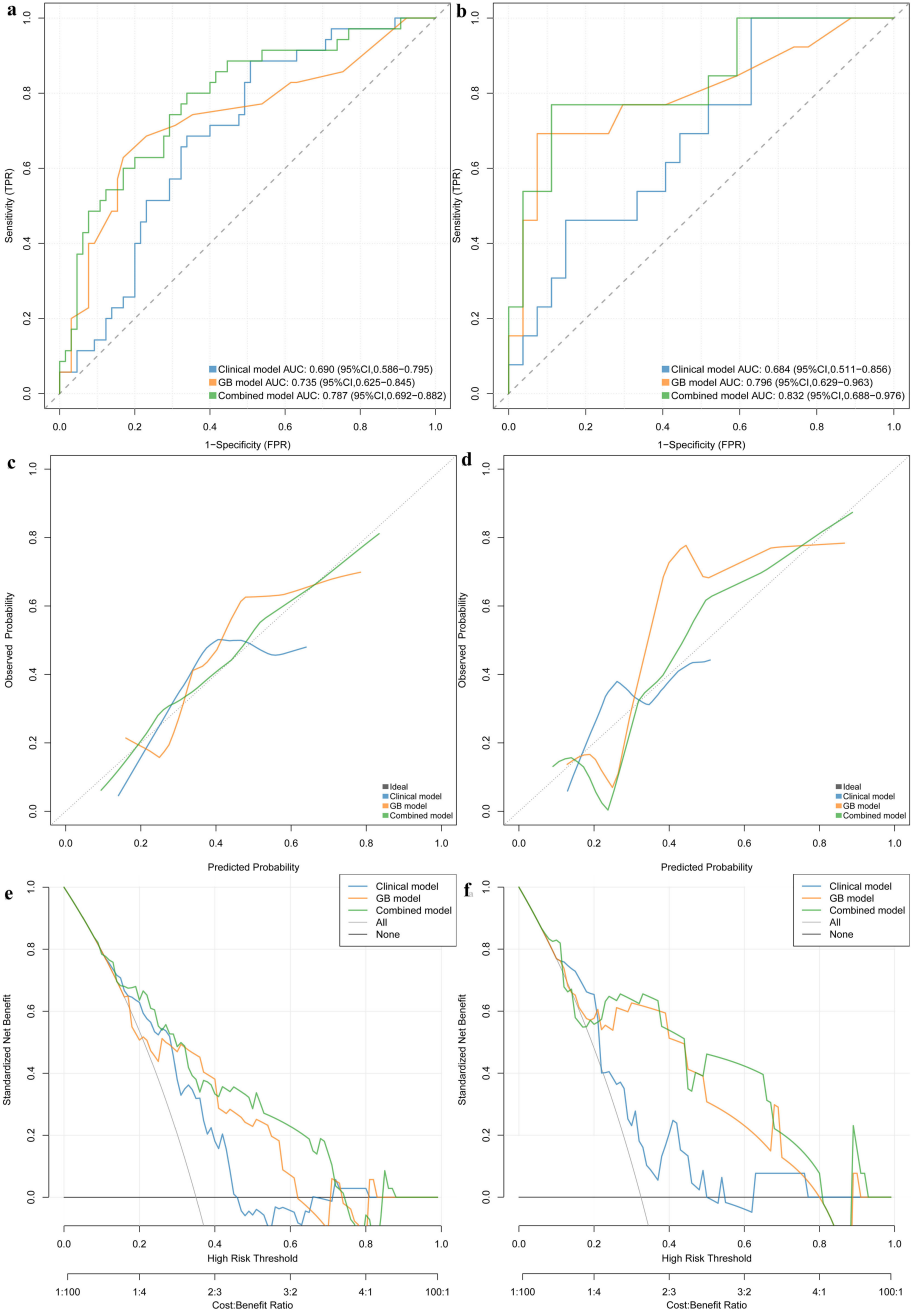
ROC curve in the training set and test sets for differentiating STEE from GBM **(A, B)**. Calibration curves of this nomogram in training set and validation set **(C, D)**. Clinical decision curve for the clinical model, TB model and combined model **(E, F)**. Based on the DCA curve depicted in **Figure 4**, it is evident that when the probability threshold ranges between 0.2-0.3 or 0.4-0.7, the green line surpasses both the blue and yellow lines, indicating a superior net benefit of the combined model compared to both the clinical and predictive models. This observation highlights the enhanced predictive efficacy of the combined model.

The nomogram, which presents the rad-score derived from TB model along with clinical valuables, is illustrated in **Figure 3**. The performance of the clinical model, TB model, and combined model is shown in **Figure 4**. The findings demonstrated that the combined model exhibited AUC values of 0.787 and 0.832 in the training and test sets, respectively. The Delong test showed that the combined model exhibited superior performance in distinguishing between STEE and GBM in both the training and test sets compared to the clinical model (with p-values of 0.025 and 0.016, respectively). However, no statistically significant differences were observed between the combined model and TB models, as well as between TB models and clinical models. The calibration curve showed good agreement between the predictions and observations. The Hosmer-Lemeshow test demonstrated a satisfactory goodness-of-fit in both the training and test sets (both *P*>0.05). Moreover, the DCA based on the combined model exhibited superior performance compared to those based on clinical and TB models in distinguishing patients with STEE from GBM. In the DCA curve, the probability threshold is depicted on the horizontal axis, while the net benefit rate is represented on the vertical axis. The black horizontal line at the bottom signifies a zero net benefit rate in absence of treatment, whereas the gray curve illustrates variations in net benefit rates with changing probability thresholds under treatment. Within a specific range, higher model net benefit rates correspond to increased clinical utility. The DCA curve in **Figure 4** demonstrates that within a wide threshold range, the net benefit of the combined model surpasses that of both the clinical and predictive models, thereby indicating a higher predictive efficacy for the combined model. As can be seen from the DCA curve, when the green line is higher than the blue and yellow lines, the net benefit of the combined model exceeds that of the clinical model and the prediction model, indicating that the combined model has a higher prediction effect.

## Discussion

Previous studies have demonstrated that the 5-year survival rate for ependymoma grade 2 and grade 3 is approximately 90% compared to 68% (28), whereas patients diagnosed with GBM typically exhibit a median survival of merely 15 months (29, 30). Given their different prognoses, accurate preoperative diagnosis is crucial in determining appropriate treatment options for patients with these different types of brain tumors. In this study, we conducted a comprehensive performance comparison of multiple machine learning algorithms (RF, LASSO, SVM, ANN, GB, TB and PLS) and developed a nomogram to differentiate between STEE and GBM. The results of our study demonstrated that the TB algorithm outperforms the other six algorithms in discriminating these tumor types. Furthermore, the pipeline yielded a more robust model with nine statistically significant features. Moreover, age, tumor size, and rad-score emerged as the most influential factors for distinguishing between these two types of tumors. Importantly, the nomogram incorporating rad-score and clinical features demonstrated superior discriminatory capacity in identifying STEE and GBM tumors.

According to the 2021 World Health Organization (WHO) CNS Classification of Tumors, ependymomas are subdivided into 10 diagnostic categories that accurately reflect prognosis and biological features based on histological, molecular information and site of onset. For any subtype of ependymoma, maximum surgical removal is one of the most important factors for optimal treatment results. However, in glioblastoma, surgery is used as the preferred treatment, supplemented by radiation therapy and chemotherapy. Therefore, accurate differentiation between the two conditions is crucial prior to surgery in order to formulate a rational treatment plan and enhance the prognosis. Several studies have documented the MRI features of extraventricular ependymoma, which can present as a voluminous mass resembling an aggressive GBM. Typical MRI findings for ependymoma include the presence of varying-sized cysts, necrosis, hemorrhages, and calcifications, accompanied by moderate or mild peritumoral edema and marked heterogeneous contrast enhancement (31, 32). However, these findings alone are insufficient for comprehensive characterization of these tumor subtypes.

Despite the significant potential demonstrated by recent advancements in artificial intelligence (AI) for enhancing brain tumor classification accuracy, there is a paucity of reports on the identification of ependymoma and high-grade gliomas, including GBM. According to a study conducted by Yao et al., the combination of six Visually Accessible Rembrandt Images (VASARI) features and four clinical features was identified as the optimal predictor for intracranial extraventricular ependymoma (IEE) and GBM, exhibiting AUC values of 0.99 and 0.97 in both the training set and validation set (33). Additionally, Safei et al. utilized SVM, RF, and LASSO models to construct a radiomic signature that effectively discriminated between STEE and high-grade gliomas. The achieved accuracy was 68% in the training set, exceeding 80% in the validation set, with an overall specificity of more than 90% (34). Furthermore, Li et al. showed that seven independent predictors extracted from VASARI exhibited an AUC exceeding 70% in discriminating between IEE and GBM (34). Despite the promising findings from the aforementioned AI studies, there are notable disparities in the results obtained for distinguishing between these two tumor types. This highlights the intricate nature and formidable challenge associated with employing AI to accurately discriminate between distinct tumor categories. Consequently, this study aims to differentiate STEE from GBM by utilizing a nomogram.

SVM, LASSO and RF are the most widely used machine learning algorithms today across various domains. However, few studies have analyzed other machine learning algorithms in comparison. In this study, we further incorporated three algorithms (ANN, TB, GB, and PLS) to conduct a comparative analysis of their performance in relation to the traditional algorithms. The implementation of these novel algorithms enables a more comprehensive evaluation of the performance discrepancies among various approaches in discriminating between STEE and GBM. The results demonstrated that TB model exhibited robust performance in discriminating the diagnostic efficacy between STEE and GBM, with an AUC of 0.735 and 0.796 in the training set and test set, respectively. Among them, TB uses gradient lifting technology to effectively solve the problem of data imbalance, and its advantage lies in its ability to model complex relationships in data sets. In addition, it also has a good performance in feature selection, which can automatically select the best partition feature and prune to avoid overfitting problems. However, unfortunately, the TB algorithms did not exhibit significant improvement in terms of diagnostic performance compared to the aforementioned studies. The potential discrepancy between the VASARI algorithm itself and the TB algorithm may account for this observation. Additionally, there may be limitations in the quality and quantity of data available for training AI models. The accuracy and reliability of AI predictions heavily rely on having access to comprehensive datasets that encompass a wide range of tumor samples representing various subtypes. If there are biases in the data used for training, it can lead to inconsistent results when attempting to classify tumors. Furthermore, variations in imaging techniques and protocols across different medical institutions can also contribute to disparities in AI-based tumor classification. Different imaging modalities may capture distinct features or provide varying levels of detail, which could impact the performance of AI algorithms trained on specific datasets.

Further analysis revealed that age, tumor size, and rad-score were identified as the most significant factors for distinguishing between these two tumors. The observation suggested that the size of the tumor and the age of patients played a significant role in determining the likelihood of receiving a diagnosis of STEE or GBM. Younger patients with smaller tumor volumes were more likely to be diagnosed with STEE within the given context. It is worth noting that no significant difference was observed in the involvement of lateral ventricles between STEE and GBM. The fact that STEE originates from ependymal cells near the lateral ventricle makes this region particularly susceptible to tumor development. Similarly, GBM also has a high incidence rate in this area due to its abundance of glial stem cells, which have the capacity for self-renewal and differentiation into GBM (20, 35, 36). These findings highlight the importance of considering patient age and tumor volume when diagnosing STEE or GBM.

The nomogram, serving as a comprehensive multivariable visual prediction model incorporating multiple variables, facilitates the holistic consideration of various factors’ impact on outcomes and is well-suited for assessing radiomic characteristics and clinical risk factors in decision-making processes (37–39). In recent years, the nomogram has gained widespread usage in quantifying risks based on diverse significant and independent prognostic factors associated with malignant tumors. In our study, we conducted a comprehensive analysis to compare the combined model, TB models, and clinical models in terms of their efficacy in identifying patients with STEE and GBM. We hypothesized that integrating rad-score and clinical features into a nomogram would result in superior predictive performance compared to individual models. Interestingly, our observations revealed that the nomogram exhibited superior performance compared to the clinical model, thereby demonstrating its superiority. Remarkably, when evaluating the discriminative ability between both tumor types using the TB model and comparing it with that of the nomograms, comparable performance was observed. However, both training and test sets in the nomogram demonstrated an increase in AUC values to 0.787 and 0.832 respectively, indicating enhanced accuracy and reliability. Clinical factors may demonstrate comparable efficacy to machine learning algorithms in disease prediction; however, clinical models often rely on a limited number of variables or indicators for decision-making and are susceptible to subjective influences and inherent limitations. A nomogram or machine learning algorithm can utilize this data to capture significant and pertinent information, which can then be combined with statistical analysis and prediction. Based on the DCA curve depicted in **Figure 4**, it is evident that when the probability threshold ranges between 0.2-0.3 or 0.4-0.7, the green line surpasses both the blue and yellow lines, indicating a superior net benefit of the combined model compared to both the clinical and predictive models. This observation highlights the enhanced predictive efficacy of the combined model.

There are several limitations in our study that warrant acknowledgment. Firstly, the small sample size may restrict the generalizability of our findings. Additionally, the absence of data support from multi-center studies limits the robustness of the model. Multicenter studies would yield more robust results and facilitate subgroup analysis to explore potential confounding factors. Finally, future studies should incorporate more advanced MRI techniques such as arterial spin labeling (ASL) and amide proton transfer (APT), which can provide valuable insights into the internal microstructure of tumors and potentially enhance the accuracy of the predictive model.

In conclusion, the machine learning-based nomogram provides a non-invasive approach to differentiate patients with STEE from those with GBM.

## Data Availability

The raw data supporting the conclusions of this article will be made available by the authors, without undue reservation.
